# Silicon Nanowires Synthesis by Metal-Assisted Chemical Etching: A Review

**DOI:** 10.3390/nano11020383

**Published:** 2021-02-03

**Authors:** Antonio Alessio Leonardi, Maria José Lo Faro, Alessia Irrera

**Affiliations:** 1 Dipartimento di Fisica e Astronomia “Ettore Majorana”, Università di Catania, Via Santa Sofia 64, 95123 Catania, Italy; antonio.leonardi@dfa.unict.it (A.A.L.); mariajose.lofaro@dfa.unict.it (M.J.L.F.); 2 Consiglio Nazionale delle Ricerche—Instituto Processi Chimico-Fisici (CNR-IPCF), Viale F. Stagno D’Alcontres 37, 98158 Messina, Italy; 3 Consiglio Nazionale delle Ricerche—Istituto per la Microelettronica e Microsistemi (CNR-IMM) UoS Catania, Via Santa Sofia 64, 95123 Catania, Italy

**Keywords:** silicon, silicon nanowires, MACE metal-assisted chemical etching, nanotechnology, CMOS-compatible

## Abstract

Silicon is the undisputed leader for microelectronics among all the industrial materials and Si nanostructures flourish as natural candidates for tomorrow’s technologies due to the rising of novel physical properties at the nanoscale. In particular, silicon nanowires (Si NWs) are emerging as a promising resource in different fields such as electronics, photovoltaic, photonics, and sensing. Despite the plethora of techniques available for the synthesis of Si NWs, metal-assisted chemical etching (MACE) is today a cutting-edge technology for cost-effective Si nanomaterial fabrication already adopted in several research labs. During these years, MACE demonstrates interesting results for Si NW fabrication outstanding other methods. A critical study of all the main MACE routes for Si NWs is here presented, providing the comparison among all the advantages and drawbacks for different MACE approaches. All these fabrication techniques are investigated in terms of equipment, cost, complexity of the process, repeatability, also analyzing the possibility of a commercial transfer of these technologies for microelectronics, and which one may be preferred as industrial approach.

## 1. Introduction

In the last half-century, microelectronics and telecommunications have completely changed our world. The two application fields have in common the use of silicon (and silicon oxide) technology for both integrated circuits and optical fibers. The huge abundance on earth combined with its low-cost and its good electrical properties made the Si extremely advantageous compared to other semiconductors. Moreover, the stability, the easy and finely controlled realization of silicon oxide have determined the arising of Si as the leading material of the current technology, even outstanding germanium. Since 1970, we saw the doubling of Si transistors inside the same integrated circuit area approximately every 2 years. This technological trend was discovered by Moore, and is known as Moore’s law [1,2,3]. However, the trend has started to change in the last decades, reaching the saturation regime due to the complexity of a further down-scaling.

Nowadays, one of the strictest limits is represented by the interconnection bottleneck. The dimension decrease implies longer and so, more power- and time-consuming interconnections, in addition to a more complex circuit realization [4]. The interconnection bottleneck due to RC delays limits the advantages of device downsizing, hindering their further downscaling. These issues are not the concern of a future challenge but are already present today. 

**Figure 1:**
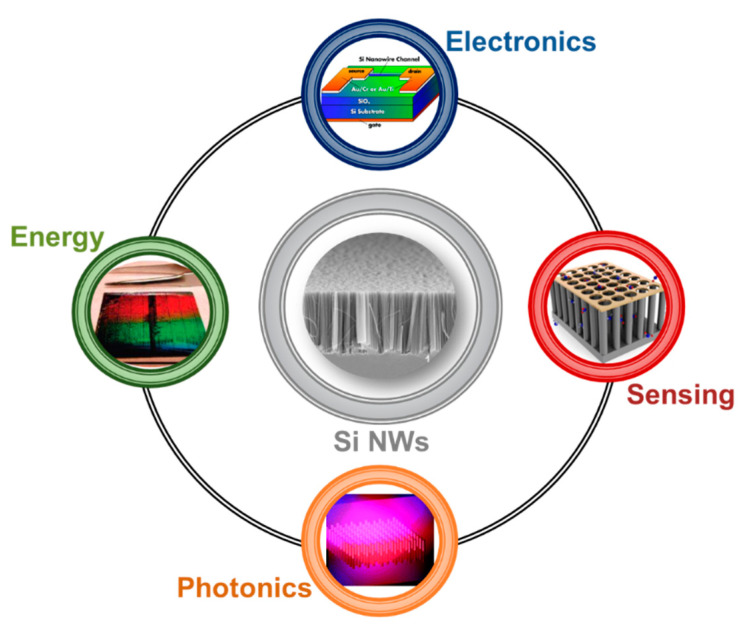
Schematic showing the main application fields of silicon nanowire from the top corner to the left one in clockwise order: Electronics with a Si NW Field-Effect Transistor [7], Sensing with a Si NWs-based gas sensor [16], Photonics with Si NW luminescence [11], Energy with a solar cell based on a vertically-aligned Si NWs array [8]. Top [7] and right [16] images are reproduced with permission, Copyright 2005 and 2011, IOP Publishing. Bottom [11] and left [8] images are reproduced with permission, Copyright 2010, American Chemical Society.

The arising of new physical phenomena on the nanoscale promoted the emerging of Si nanostructures for the past, present and future technologies. In particular, silicon nanowires (Si NWs) developed as a novel resource in many different fields, such as electronics [5,6,7], photovoltaics [8,9,10], photonics [11,12,13], and sensing [14,15,16], as schematized in Figure 1.

Indeed, 1D nanostructures can be easily integrated in the typical flat architectures of integrated circuits, benefitting from such nanomaterials’ advantages.

During these years, a lot of effort was spent on the realization of novel Field Effect Transistor (FET) based on Si NWs [17,18,19], as well as on the integration of silicon photonics in microelectronics industries [11,20,21]. Metal Oxide Semiconductors Field Effect Transistor (MOSFET) technology’s constant miniaturization for microelectronics led scientists to design new improvements based on nanomaterials, such as nanowires and nanotubes. Si NWs arise as a natural candidate, and Lieber’s group [5] showed their substantial advantages on the state-of-the-art of planar silicon FET transistors. Their Si NW FET was obtained using the same planar geometry on a silicon bridge between the source and the drain. The FET was realized with p-type Si NWs having diameters of 10–20 nm dispersed on a 600 nm silicon oxide layer on top of a silicon wafer. Moreover, the paper of Feng et al. [6] reports a low-frequency noise behavior of Si NW FET compared to the planar standard due to the electrons quantum confinement in 1D. As an example of a typical Si NW FET architecture, the device obtained from Koo et al. [7] is shown at the top of Figure 1.

In the list of our time’s most critical challenges, the energy demand and climate changes are becoming more important every day. Climate changes are strictly related to the energy and electricity production methods and uses [22]. To prevent a disastrous scenario, it is a priority to reduce CO emissions by 75% by 2050 and converting to renewable energy production is now mandatory. Photovoltaics is a strategic alternative to address this challenge compared to other sources that has become cost-competitive for the energy market also taking advantage of the cheap silicon manufacturing [23,24].

Si nanowires are of interest by improving the light trapping inside the solar cell with a lower thickness of at least 2 orders of magnitude. Another advantage of Si NWs is the possibility to realize radial p-n junctions, drastically reducing carriers lost. Atwater’s group [9] demonstrated the realization of a solar cell where the p-n junction is formed by embedding the p-type 2–10 μm long Si NWs into a polydimethlydiloxane (PDMS) layer (a conductive polymer). Si NWs were synthesized by VLS covered by 80 nm SiNx anti-reflective coating, embedded into the PDMS containing Al_2_O_3_ particles (0.9 μm of diameter) to further improve the performances with a peak external efficiency of 0.89. Garnett et al. [8] demonstrated substantial advantages in the use of Si NWs. Silicon NWs are realized by using Deep Reactive Ion Etching (DRIE) with a silica beads monolayer as the mask. Silicon is etched in the area where the silica beads are not present and 5 µm long Si NWs are obtained. Finally, the silica beads are removed by a hydrofluoric acid etching. To obtain the radial p-n junction, a boron doping of the starting n-type Si NWs was realized, as shown in the left part of Figure 1. By this strategy, the light path length inside the solar cell is increased by a factor of 73 with 10 times higher efficiencies than by using a thin silicon layer with the same thickness of the Si NWs, with final efficiency of 5%, probably due to the recombinations. 

The use of light in plenty of fields, from intercommunications to energy production and medicine, changed our life. One of the most interesting challenges is light to transport information inside an integrated circuit for Si microphotonics. Despite all the silicon advantages, it is an indirect bandgap semiconductor making the integration of light signals in a silicon platform a hard and still open challenge. In the photonics field, the application of silicon nanowires is extremely limited due to the diameter required to observe light emission by quantum confinement effect. Indeed, the quantum confinement effect requires diameters under 10 nm [25,26,27] that are extremely complex to obtain with standard approaches, such as Vapor-Liquid-Solid (VLS) or even very expensive Electron Beam Lithography (EBL) processes coupled with DRIE [11]. A strategy used in literature is the successive oxidation of Si NWs to achieve quantum confinement suitable diameter. The group of Brongersma demonstrates a tunable photoluminescence (PL) emission from Si NWs fabricated by Ti-catalyzed VLS and further oxidated at 950 °C in O_2_ and then in Ar [28]. Walavalkar et al. fabricated an ordered array of Si NWs by EBL and DRIE with a starting diameter of 20 nm and further oxidized in a dry ambient in the temperature range of 850–950 °C [11]. This method is more expensive than the previous one. EBL limits a large-scale production, even if novel approaches as NIL promise to solve this issue. A common drawback for both these cases is that a thick oxide layer completely passivates the Si NWs making very complex electrical pumping. At the bottom of Figure 1, the photoluminescent Si NWs realized by Walavarkar et al. [11] are reported as an example for the photonics field. 

Silicon nanowires are strategic solutions even as low-cost and highly sensitive sensors demanded by our society for the early screening of pathologies [29]. However, Si-nanostructured sensors remain scarcely diffused due to their expensiveness and their incompatibility with CMOS technology [30]. In this field, Si NW Field-Emission-Transistor sensors enabled single virus sensitivity detection [15,31]. In the right part of Figure 1, the Si NWs-based sensor obtained by In et al. [16] is shown as an example of sensing applications. However, these sensors are realized by using cutting-edge lithographies (as EBL) and etching approaches, making the platform realization still too complex and expensive for a real commercial transfer. 

The approaches used for Si NWs realization have a crucial impact on all these application fields influencing the performances and the cost of the final devices. Standard methods such as VLS and Reactive-Ion Etching (RIE) or DRIE coupled with advanced lithography present several issues that can negatively affect the use of Si NWs in all these applications. In this scenario, Metal-Assisted Chemical Etching (MACE) arose as an innovative synthesis method able to couple a CMOS cost-effective synthesis with a large-scale and microelectronics compatible fabrication. The VLS and RIE/lithography drawbacks will be highlighted in the next paragraph and compared with the characteristics of the MACE. The main MACE approaches for the Si NWs synthesis will be highlighted and critically discussed giving an overview of the main advantages and drawbacks of each one of the presented strategies. In this work we will give an overview on both single step and two step MACE approach reporting all the main synthesis parameters that can permits to use this approach for both vertically aligned, porous, tilted, or kinked Si NWs and microstructures fabrication. Moreover, in this review we will report also novel approaches able to realize ultrathin Si NWs with a large scale and industrial compatible approach.

## 2. State of Art of Si NW Synthesis 

**Figure 2:**
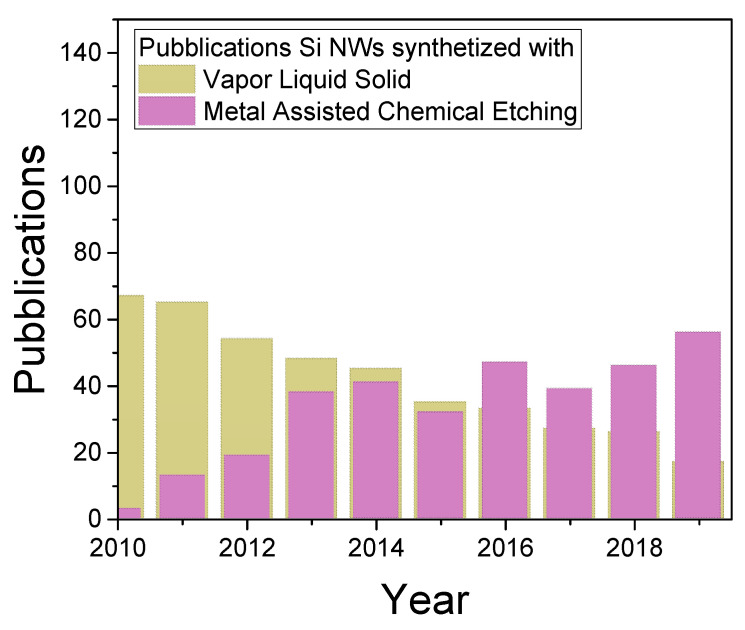
Bibliometric analysis of Metal Assisted Chemical Etching compared to Vapor Liquid Solid for the fabrication of Si NWs.

Vapor-Liquid- Solid has been considered for a long time since its discoveries in 1964 by Wagner et al. the main method for the cost-effective and large-scale Si NW fabrication [32]. However, VLS has become to be surpassed by the Metal-Assisted Chemical Etching, as attested by the bibliometric trend over the last 10 year reported in Figure 2. The data were obtained from ISI Web of Science Database by using “silicon nanowire” and (logic and) “Vapor Liquid Solid” or “Metal Assisted Chemical Etching” as topics. As it can be observed from the graph, there is a trend change between the two approaches. This could be ascribed to the VLS several drawbacks that can be surpassed by MACE. In this manuscript, VLS, RIE, and different lithography approaches will be presented, providing an honest comparison among the possible MACE approaches. In particular, the advantages and drawbacks of VLS and Reactive-Ion Etching will be discussed, with an insight on their combination with lithographies. 

### 2.1. Vapor-Liquid-Solid Approach

Vapor-Liquid-Solid has been for a long time the most diffused approach for the Si NWs fabrication. Despite its strong diffusion, this approach suffers of several drawbacks that strongly limited the applications of the synthesized Si NWs [33,34]. The growth of NWs is catalyzed by metal droplets that are realized as a product of the melting of Au nanoparticles (NPs) contaminated with silicon atoms [35,36]. The most used catalyst remains the gold due to the high crystalline quality of the realized Si NWs and the simple thermodynamics physics of the Si/Au alloy. These droplets of gold-silicon alloys are liquid at the eutectic phase that is obtained through specific growth conditions. When the silicon concentration inside the alloy becomes higher than the Si concentration at the thermodynamic equilibrium of the eutectic phase, the silicon precipitates. Hence, Si precipitates under the gold, solidifies, and grows as nanowires [35,36,37]. One of the most important advantages of the VLS is the flexibility of the approach that can be adapted to different equipment and approaches such as Chemical Vapor Deposition (CVD), Thermal Annealing, Thermal Evaporation, Molecular Beam Epitaxy (MBE), Pulsed Laser ablation, and Chemical Bath Deposition (CBD) [35]. VLS offers high flexibility over the Si NW growth rate from 10^−2^ to 10^3^ nm/min [38,39]. Another advantage is the possibility to obtain Si NWs with a diameter down to about 10 nm as shown by Lieber’s group [5] and even below this limit. Recently, Puglisi et al demonstrated the realization of Si NWs with a diameter under 10 nm by varying the plasma power and the gas pressure in a VLS process realized by a plasma enhanced chemical vapor deposition [40]. However, their synthesis is still complex and the Si NWs showed in these papers have different orientations making more complex their implementation for real applications. Hence, even if it is possible to reach less than 10 nanometer in diameter, it leads to a lack of orientation control of the Si NW growth [40,41].

The NW diameters obtained in most of the works were always higher than the Au NPs ones. In the work of Hochbaum, a maximum density of 1.8 × 10^8^ NWs/cm^2^ is obtained [42]. Commonly, higher densities are difficult to obtain by VLS due to the metal NPs arduous manipulation. Moreover, due to the high temperature of the process, Ostwald ripening occurs and the agglomeration of small particles into bigger ones is thermodynamically favorable. These phenomena make the deposition of metal nanoparticles very laborious in terms of positioning and preparation of the substrate. Another strong constraint to the NW diameter is due to the Gibbs-Thomson effect [36] that limits the radius on the order of 10 nm for the growth of Si NW catalyzed by gold.

The high thermal budget required determines several problems, such as a gold diffusion inside the Si NWs [43] that represent a crucial limitation. Due to the high temperature required by the VLS synthesis process, the gold diffuses inside the Si NWs introducing new trap levels in the middle of the bandgap. As a consequence, non-radiative recombination due to the Shockley-Read-Hall (SRH) effect became more efficient. This effect dramatically affects the performance of the Si NWs in electrical [34] as well as optical applications [44].

The doping is commonly realized during the growth by the use of another gas precursor containing the dopant species. However, due to the high temperature a disuniform radial doping profile is obtained. Koren et al. demonstrate a disuniform profile of doping [45]. To surpass this drawback, other doping methods are used in literature as low energy ion implantation. However, in this case, several defects are introduced to the silicon nanowires [17] and in some cases can be even present a plastic deformation of the Si NWs due to the amorphization of the silicon [46]. Even with other approaches that can solve these drawbacks (as molecular doping [47]), a further doping process is required making the synthesis procedure more complex and expensive.

Several other catalysts were studied in the literature [35] permitting to overcome the massive presence of SRH recombination obtained with gold but without solving the other issues of this approach. However, in most cases metal contamination is still present due to the high required temperature. In the best-case scenario for certain catalysts as Al or Ga [35], this is traduced in uncontrolled slight doping for the final wires. However, using metal different from Au commonly a final lower quality of the fabricated Si NW array is obtained [35]. 

### 2.2. Reactive-Ion Etching and Lithography Approaches

RIE is a CMOS compatible top-down approach. In particular, this method is a dry etching approach based on a directional etching by a reactive ion plasma. A strong radio frequency (RF) electromagnetic field generates the plasma of the reactive gas in a low-pressure chamber and the ions are accelerated to the sample. By using an RF electromagnetic field, the electrons are in each cycle accelerated much more than the ions due to the huge difference of mass. When the electrons reach the chamber wall are eliminated by the ground of the chamber. However, the electrons reaching the sample (that is electrically isolated from the rest of the chamber) charge it negatively, attracting the plasma ions to the sample. An evolution of this process is the DRIE, one of the most used etching approaches to obtain very high aspect ratio structures. Differently from standard RIE, in this process, a cycle of RIE etching is followed by passivation of the sample, and these two steps are repeated several times. In this way, the effect of the ion direction plays a greater role than in the standard etching, obtaining a higher anisotropic etching and smoother walls. RIE and DRIE etching rates are determined by the chemical affinity of the reactive ion gas to the material. Different materials require the use of different gases to be etched. This can be used to etch a specific material on a substrate without etching the entire substrate. Indeed, to realize a specific pattern the substrate has to be masked with a material chemically inert compared to the used reactive gas. Several approaches can be carried out to mask the substrate, from cost-effective self-assembly methods to more complex and expensive lithographies.

The coupling of DRIE with advanced lithography approaches has been deeply employed for the realization of Si NWs. Indeed, in several papers EBL is used for Si NW fabrication [35,48]. Due to the high aspect ratio, this approach is suitable for the realization of Si NWs surpassing several drawbacks of the VLS approach such as impurity levels and doping. The etching is generally obtained by using SF_6_ or chlorine (Cl_2_) gas to selectively etch the silicon and a C_4_F_8_ passivation gas to enhance the anisotropic effect. Suitable materials, used as a specific mask for the etching of Si, are Ti, Cr, or even polystyrene particles [8,49] that are not or only slightly etched during this method. With this approach, diameters on the order of tens of nanometers can be obtained. However, this limits the length of the synthesized Si NWs. Indeed, a common limit in the aspect ratio of about 50:1 (in some cases up to 100:1) [50,51,52] strictly correlates the diameters to the final achievable lengths of the Si NWs. However, the realization of diameters on the orders of tens of nanometers is extremely complex. Besides, Si NWs realized with these approaches have a rough surface and in several cases, even a damaged surface that induces recombination losses. This is a crucial drawback for the realization of any type of device.

The use of self-assembly methods is a powerful and cheap resource for the masking procedure. Approach as de-wetting through thermal annealing process or Langmuir-Blodgett were successfully addressed in several applications. However, there are cases where a stronger control over the device features is required and in these cases, other techniques need to be carried out. UV lithography is used every day in the current CMOS industry to realize complex integrated circuits on top of a Si substrate [53]. UV Lithography permits to have a strong control up to the limit of UV light diffraction corresponding to hundreds of nanometers [54,55]. The recent improvement of this approach through the use of a UV laser is known as extreme UV lithography and promise in the next few years to push this limit under tens of nm with an industrial compatible approach [56,57]. In all the cases, a UV source is used to change the local property of a polymer layer to produce a mask for further material deposition (i.e., Au NPs for VLS) or selective etching (i.e., RIE for Si etching). The UV light is used to change the solubility of the resist concerning a specific solvent called resist developer. The minimum feature achievable by UV lithography is proportional to λ/NA, where λ is the wavelength used and NA the numerical aperture of the lens. So, in principle, by decreasing λ and decreasing the beam spot (increasing NA), it is possible to push down this limit. However, other limitations related to the focus of the beam are also present. To surpass the diffraction limit of the UV light, several approaches were proposed as X-ray lithography [58], nanoimprinting lithography [59], and Electron Beam Lithography [60,61]. 

EBL is the most used approach compared to the others due to its flexibility of use. In an EBL process, electrons are used instead of photons with the advantage of a shorter wavelength. In fact, EBL be carried out by using a standard Scanning Electron Microscope (SEM), whose typical energy spans from 100 eV to 30 KeV with associated λ from about 12 nm to 0.04 nm, respectively. Another advantage is the fact that the electron beam is directly focused and driven to span the polymer with a chosen pattern without the need of a mask, required in other approaches such as conventional UV Lithography or NIL. However, the use of an SEM as EBL equipment permits to achieve a maximum resolution of several tens of nanometers (commonly on the order of 50 nm). To push down this limit to sub-10 nm, more expensive and dedicated equipment are required [62] achieving a limit of about 5 nm [63]. Indeed, this method is strongly limited with respect to the total writing area achievable, which commonly is on the order of few centimeters, making it unsuitable for large scale production and industrial processing.

Nanoimprinting Lithography is based on the mechanical deformation of the resist that subsequently is processed through UV light exposure (photo NIL) or by heating (Thermoplastic NIL). A nanoscale patterned mold is used to mechanically deform a spun polymer by using a thermal or cold-welding process. The polymer is then cured by light (photo NIL) or by either a thermal or cold-welding process (Thermoplastic NIL). This approach is in general cheap and CMOS compatible with the possibility of large-scale applications. However, NIL results still limited for the feature dimension achievable on tens of nanometers and further improvements are required to reach the resolution limit of EBL. 

In conclusion, despite EBL is not suitable for large-scale production, in the last years other lithography strategies emerge as a viable route. For example, nanoimprinting lithography seems to be able to replace EBL with similar features permitting a large-scalable fabrication. However, the high cost, the need of expensive dedicated equipment, and the limit of 50:1 on DRIE aspect ratio determine the difficulty to achieve nanometer diameters (e.g., for quantum confined) and high-density Si NWs arrays. However, the use of lithography remains interesting even coupled with other etching procedures. Indeed, a lot of effort was spent in the literature on the realization of ordered structure of Si NWs and these techniques can be integrated with a bottom-up, as well as in a top-down process as the MACE [64]. 

### 2.3. Metal Assisted Chemical Etching Approach

**Figure 3:**
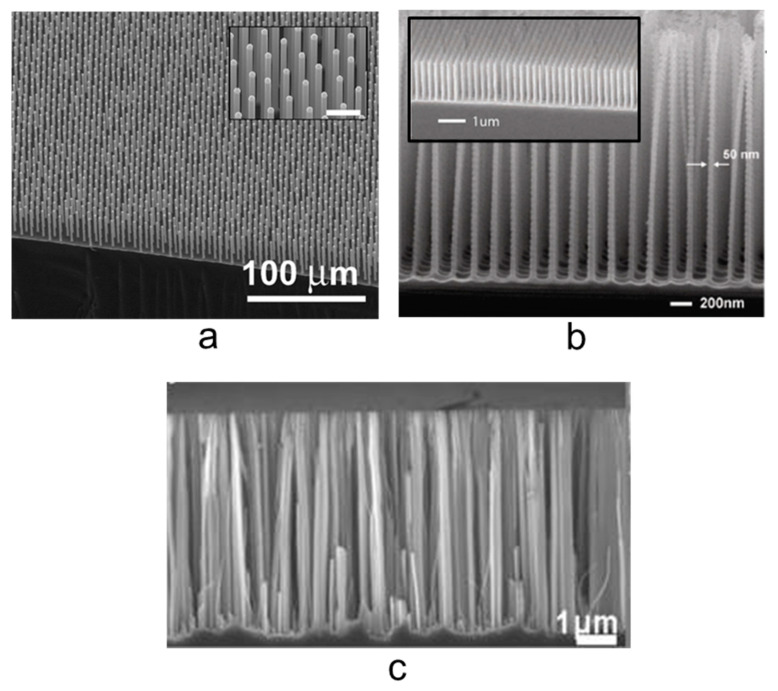
Cross-section SEM images of Si NWs synthesized by (**a**) VLS [65], (**b**) RIE coupled with NIL [66], and (**c**) silver salt MACE [67]. In the inset to (**a**) a higher magnification is reported with a 10 µm marker. In the inset to (**b**) a tilted image that shows the density is visible. (**a**) ref. [65] is reproduced with permission, Copyright 2007, AIP Publishing. (**b**) ref. [66] is reproduced with permission, Copyright 2008, IOP Publishing. (**c**) ref. [67] is reproduced with permission (open access), Copyright 2011, Springer Nature.

MACE approach is based on a wet etching process driven by a metal catalyst deposited onto the surface of the silicon. The process is typically at room temperature and no metal contamination is attested in the final nanostructures. In Figure 3 is reported the comparison among different cross-section SEM images obtained for Si NWs realized by several methods. In Figure 3a a Si NW array obtained by VLS through a CVD process using SiC_4_ as precursor gas by Kayes et al. is reported [65]. In the inset to 3a is shown a high magnification of the precedent SEM image with a marker of 10 µm. Figure 3b reports the Si NWs obtained by Morton et al. by a NIL and DRIE process [66]. As described during this paragraph, a higher roughness of the walls is attested. In the inset to Figure 3b a tilted image that shows the density is visible. Finally, in Figure 3c the Si NWs array fabricated by the standard silver salt MACE by Nassiopoulou et al. is shown [67]. MACE permits to obtain a higher density of Si NWs with smoother walls. Without any type of masking procedure, the array results disordered. In the next paragraphs, detailed analyses of some of the most interesting MACE approaches will be presented. 

MACE is an anisotropic wet etching that uses high electronegative metal to catalyze and drive the etching process. This etching method was proposed and demonstrated for porous silicon fabrication for the first time in 2000 by Li and Bohn [68]. 

**Figure 4:**
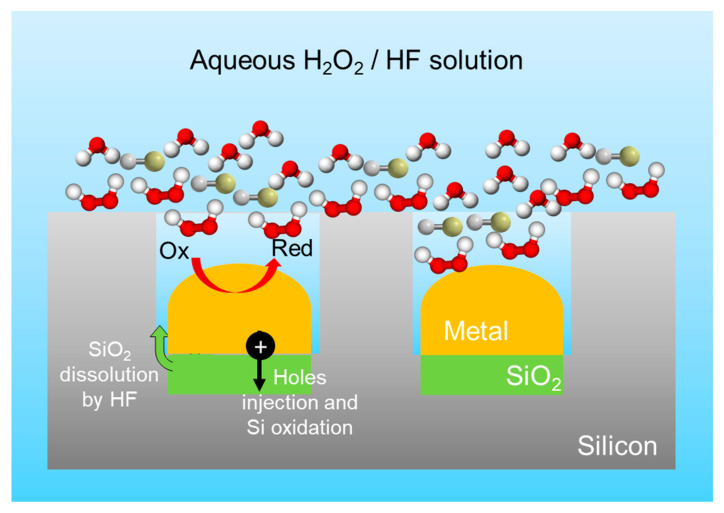
Schematized MACE etching process for a H_2_O_2_/HF aqueous solution.

To give an insight of this etching approach we can analyze the case of H_2_O_2_/HF reaction. This was the first adopted chemical solution and continues to be used in several works. We can schematize the MACE process as visible in Figure 4. 

The Si substrate is in contact with an isolated metal cluster and is etched in an aqueous solution of HF and H_2_O_2_. The chemical reactions occur at the interface between the Si and the metal. In this approach, we can consider the Si as a local anode with the metal acting as a cathode for the current produced in the Si/metal interface. 

The kinetic of the process is the following:

(1) $$H_{2}O_{2}\;+\;2H^{+}\;\to \;2H_{2}O\;+2h^{+}$$

As suggested by the same fathers of this approach, Li and Bohn [68], and by other different studies [69], the cathode reaction happens as the usual reduction of protons into hydrogen:

(2) $$2H^{+}\;\to \;H_{2}↑\;+2h^{+}\;\;\;$$

The reduction of the oxidant species generates holes that are injected inside the silicon underneath the metal. Hence, in the Si anode region, the silicon is oxidized and dissolved. Three Si dissolution processes were proposed by the scientific community [70]. However, up to now, none of them was experimentally demonstrated to work in-situ or to be favorite among the others. The dissolution models follow as the reaction I, II, and III, respectively.

Reaction I (RI):

(3) $$Si\;+\;4h^{+}+4HF\to \;SiF_{4}\;+4H^{+}\;$$

(4) $$SiF_{4}\;+\;2HF\to \;H_{2}SiF_{6}$$

Reaction II (RII):

(5) $$Si\;+4HF_{2}^{-}\to \;SiF_{6}^{2-}\;+2HF+H_{2}↑+\;2e^{-}$$

Reaction III (RIII):

(6) $$Si+2H_{2}O\;\to SiO_{2}+4H^{+}+4e^{-}$$

(7) $$SiO_{2}+6HF\;\to H_{2}SiF_{6}+2H_{2}O$$

In the RI case, the Si is directly dissolved in a tetravalent state without forming the silicon dioxide. In the RII the direct dissolution of Si is still present but in the divalent state. In this third model, the Si atoms in contact with the metal are oxidized and then dissolved in two different processes [70]. RI differs from the other two by involving a direct dissolution of Si (in a tetravalent state) with a gaseous formation without the generation of H_2_. In the other two models, the H_2_ generation is followed by the dissolution of the oxidized species Si and SiO_2_ in RII and RIII, respectively. The experimental evidence of bubble formation during the etching process seems to suggest RII as the favourable model. However, all these three models result valid and probably a combination of all of them happens. Indeed, the question if the reaction RIII happens simultaneously remains unsolved. This is due to the difficulty of an in-situ analysis of the surface state and the uncertainty in Si surface state ex-situ measurements due to the possible formation of oxide during the handling of the sample before a Transmission Electron Microscopy (TEM), or X-ray Photoemission (XPS) characterization. 

In MACE the hole injection from the metal to the silicon is well-documented as a charge transfer process necessary for the oxidation and dissolution of Si. In some cases, the silicon underneath the metal clusters may present a microporous structure due to the holes diffusion at the Si/metal interface [71]. It is worth to note that H_2_O_2_ injects holes into the Si valence band independently of the doping type and level. What changes between different doping type and level is the etching rate of the process [72]. A MACE process may result in the synthesis of porous Si with different pores dimension or in the fabrication of Si NWs. This depends on several parameters of the etching such as the electronegativity of the metal used, the concentration of the oxidant agent (e.g., H_2_O_2_), and the concentration of the etchant agent (e.g., HF) [67,73,74] as will be discussed in the next paragraphs.

## 3. Silver Salt and Single Step MACE

In 2002 Peng et al. realized a high density of vertically aligned Si NWs with the MACE by using an AgNO_3_:HF aqueous solution in a teflon-lined stainless-steel autoclave [75]. After three cleaning steps in acetone, ethanol, and diluted aqueous HF solution to remove organic grease and native oxide, the cleaned wafer was etched with 5.0:0.02 M solution of HF:AgNO_3_ and treated at 50 °C for 60 min. During the etching process, the silicon wafers showed the formation of a thick layer of Ag dendrites on top of the nanowires, which is promptly removed by nitric acid solution (70%). Indeed, due to the formation of silver precipitates and to the use of AgNO_3_, this method is commonly known as the silver salts approach and it is identified as the main single step MACE approach. 

**Figure 5:**
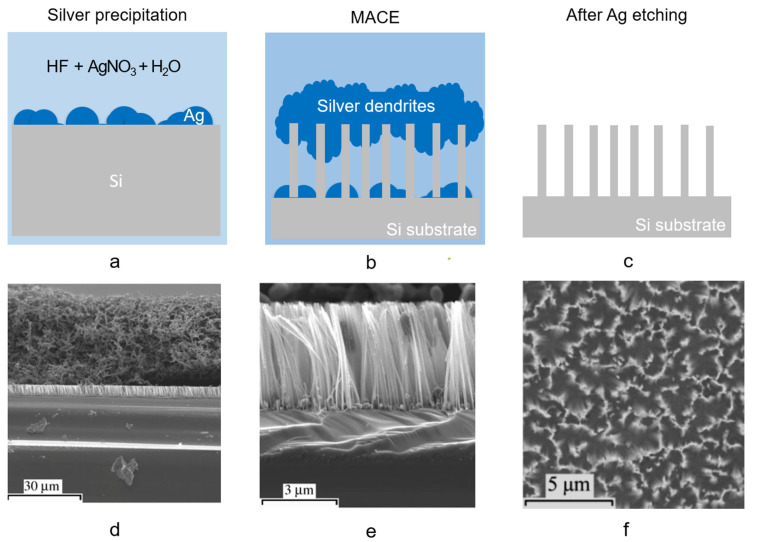
Scheme of Si NWs synthesis by silver salt single step MACE: (**a**) Ag precipitation from the HF/AgNO3 solution onto the Si surface, (**b**) Ag dendrites and Si NW formation, (**c**) Ag dendrites removal by nitric acid. Cross-section SEM of Si NW realized onto p-type (111) by HF/AgNO_3_ single step MACE for 30 min, (**d**) during Ag dendrites formation, and (**e**) after its removal [76]. (**f**) SEM plan view of Si NWs produced by HF/KAuCl_4_ synthesis after 30 s [80]. (**d**,**e**) ref. [76] are reproduced with permission, Copyright 2006, John Wiley and Sons. (**f**) ref. [80] is reproduced with permission, Copyright 2005, John Wiley and Sons.

In Figure 5a–d the scheme of the silver salt process is shown in detail. After the cleaning procedure, the sample is immersed in an aqueous AgNO_3_:HF solution (Figure 5a). When in solution, the AgNO_3_ catalyst precipitates forming Ag nanoparticles (NPs) which are randomly distributed onto the silicon flat surface, catalyzing the HF driven Si etching in a very similar way to the one described for H_2_O_2_. Indeed, AgNO_3_ acts both as an oxidant and as a metal source. Ag NPs, with dimensions and density related to the starting silver nitrate solution, precipitate onto the Si surface biasing the etching. The Ag nanoparticles formed in solution are more electronegative than the Si and inject holes into the substrate that is oxidized due to the presence of radical NO^−^_3_. The selective oxidation process is driven at the Ag NPs/Si interface resulting in the formation of SiO_2_ underneath the metal NPs, and the etching continues by the HF presence, resulting in the formation of Si nanowires in the Si uncovered regions. As a byproduct of the process, Ag dendrites are formed onto the newly etched Si NWs as depicted in the scheme in Figure 5b. The Ag dendrites form a dense network of several microns thick (about 40 µm) onto the Si NWs, as displayed in the SEM cross-section (Figure 5d). Finally, the silver dendrites in excess are selectively etched by nitric acid (Figure 5c) and the Si NWs vertical array are obtained as shown from the SEM cross-section (Figure 5e). 

Figure 5e shows the typical cross-section of Si NWs realized by using a HF/AgNO_3_ solution for 30 min with concentrations of 4.6/0.02 M. The Si NWs were obtained onto a (111) p-Si substrate preserving their crystalline quality, and are about 4 µm long [76]. The average diameter of NWs realized by single step approaches is about 70 nm ± 20 nm [77] as usually obtained by the silver salt approach, ranging from about 60 nm up to 140 nm on average [78]. As already stated, the length of the NW array can be increased with the etching time from a few hundreds of nm up to several tens of microns, leading to nanostructures with an aspect ratio above 200 [79]. Aside from AgNO_3_ precursors, also KAuCl_4_ is widely diffused for the single step MACE process. The SEM plan-view of Si NWs obtained by MACE with HF/KAuCl_4_ with concentrations ratio of 6.0/0.005 M after 30s, as shown in Figure 5f [80].

This single step silver salts MACE process is fast, does not need complicated sample preparation, and is less expensive than the other MACE procedures. In their pioneering works, Peng et al. investigated the strong correlation of the growth parameters, mainly the solution type and concentration, growth temperature, illumination, and substrate orientation. 

Indeed, a variety of oxidative metal-based solutions can be used to drive the Si etching, such as AgNO_3_ [81,82], KAuCl_4_ [80], Fe(NO_3_)_3_ [83], KMnO_4_ [84], KBrO_3_ [85], K2Cr_2_O_7_ [84], and so on, however Ag and Fe-based oxidants presents the lowest redox potential [70]. Additionally, different noble metals such as Ag, Pt, Fe, Pd, and Au are mainly used to catalytically reduce H_2_O and O_2_ [86,87,88]. In general, dendrite structures are produced during the oxidation and dissolution of the Si substrate when the metal ions are reduced to particles, resulting in the formation of vertically aligned Si NWs with differences in the oxidation and etching rate, and for the NW morphology [70]. Higher concentrations of the metal salts result in a denser metal catalyst, hence forming nanowires with lower density, smaller diameter, and more spaced within each other. Indeed, it is not simple to control the NW planar arrangement by this approach, since their diameter, density, and interspacing are determined by the metal concentration.

Another parameter highly affecting the morphologies of the etched NWs is the type of noble metal used during the MACE. Usually, Ag and Au nanoparticles formed in solution result in straight pores during the etching, while straight or helical pores can be achieved by Pt catalyst were reported by Tsujino et al. [89,90]. Indeed, through its precipitation, Pt nanoparticles move randomly during the etching, resulting in curvy pores without a uniform etching direction. Moreover, the formation of porous nanostructures is also influenced by metal. Generally, Au-coated or Ag-coated substrates result in smoother NWs with low porosity, while Pt-driven synthesis results in a more porous structure [89]. Similarly, while standard p and n doping results in a similar etching rate for the formation of low porosity NWs, the presence of a high concentration of dopants (around 10^20^ dopant atoms × cm^−3^) for p++ and n++ Si wafers results in the formation of highly porous Si NWs due to the occurrence of lateral etching. 

Peng’s MACE synthesis is a single step approach for the easy and fast formation of Si nanowires without the need of a second etching step in other oxidizing species such as H_2_O_2_ [91] or thin film depositions [92]. 

In general, this process is carried out at room temperature although the correlation between the temperature and the etching rate structure was also studied. The NW length approximately follows a linear trend with time. However, the etching rate also depends on the temperature and increases linearly between 0° to 50 °C [93]. Another parameter affecting the etching rate is illumination. If the intensity of illumination is sufficiently high so that the injection of the photoexcited holes is comparable or higher than the concentration of holes injected from the oxidant species, then the etching occurs faster. Studies revealed that the process is independent of the room illumination within a confidence of 5%, but an increment of about 1.5 times the etching rate was observed under light illumination with a 20 W lamp [94]. 

To control the axial orientation of vertically aligned Si nanowires it is possible to vary the Si wafers with other orientations, such as (100) and (110). According to the back-bond breaking theory [95,96], the (100)-orientation presents the lowest bond energy and so (100) wafer are preferentially etched vertically along with this orientation. In contrast, an atom on the (110) and (111) have three back-bond to break, hence the etching occurs again preferentially along the (100) when the etching rate dominates on the oxidation process. On the contrary, increasing the oxidant concentration favors the etching of non-(100) orientation resulting in tilted nanowires [97,98,99].

However, a crucial drawback compared to the other MACE approaches is that during the process the presence of Ag dendrites is attested onto the samples and the dendrites etching may damage the Si NWs, resulting also in Ag contaminants [100]. Hence, the MACE single step methods allow to achieve high yield, fast production rate at the expense of a lack of control on the NWs planar arrangement and diameter control [76].

Indeed, more advanced syntheses were optimized by using masked approaches, granting also the control on the NWs diameter and planar arrangement (density and spacing) by using single-step MACE coupled with lithography processes.

**Figure 6:**
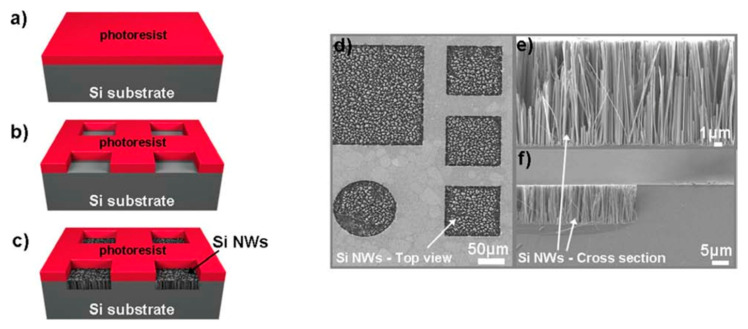
Flow chart for Si NW formation by MACE at a temperature of 30 °C on the confined areas by (**a**) applying AZ5214 photoresist, (**b**) opening windows from 2 × 2 μm^2^ to 400 × 400 μm^2^, and using (**c**) AgNO_3_/HF/H_2_O solution with a concentration ratio of 0.67 g:35 mL:182 mL. (**d**) Plan-view and (**e**,**f**) Cross-sections SEM images of the etched confined areas [67]. This figure [67] is reproduced with permission (open access), Copyright 2011, Springer Nature.

As an example, Nassiopoulou et al. used UV photolithography onto p-type (100) Si wafers with resistivity ranging of about 1 Ω × cm to open square-shaped windows ranging from 2 × 2 μm^2^ to 400 × 400 μm^2^ with the AZ5214 photoresist applied by spin coating, as shown in Figure 6a,b. Then, Si NWs were grown by MACE in a AgNO_3_/HF/H_2_O solution with a concentration ratio of 0.67 g:35 mL:182 mL at a temperature of 30 °C, as reported in Figure 6c. Figure 6d shows the top-view SEM microscopies imaged of the exposed windows where Si NWs are grown, confirming the efficiency of this approach. The SEM cross-section reported in Figure 6e,f shows that it is possible to grown 15 µm long vertically aligned Si NWs with good quality even at the window edge, confirming the robustness of two-steps MACE methods. Nonetheless, photolithography is limited to small areas of about 10^4^ µm^2^ [67], and other approaches based on two step MACE using films achieved by the metal deposition onto silica bead can also be used [101].

The primary catalysts for MACE are Ag, Au, and Pt because of their stability and catalytic activity in HF/ H_2_O_2_ solution, but apart from their high cost, the main drawbacks of these noble metals are their diffusivity in Si, which is detrimental to the performance of CMOS [102]. As an alternative W and Ni can also be used. Both W/H_2_O_2_ and Ni/H_2_O_2_ result in a lower etching rate compared to noble metal, the first one allowing the production of very large holes up to 800 nm in diameter and the latter resulting in the formation of pyramidal etched structures [103].

Gallium and Platinum ions deposited by focused ion beam can also be adopted for the Si MACE [104] and the produced NWs show poor morphology and low reproducibility. 

Ti metal was also introduced to HF/ H_2_O_2_ aqueous etchant to etch Ag-loaded Si (n-type, 100) [105], resulting in a lower etching rate by increasing Ti concentration. This decrease was attributed to the lowering of H_2_O_2_ concentration because TiF_6_^2−^ produced by the reaction of Ti and HF can complex with H_2_O_2_ to form anions of [TiF_6_(H_2_O_2_)]^2−^. 

Similar to AgNO_3_, also AgO (insoluble) [106] and Ag_2_O (soluble) [107] have been utilized instead of AgNO_3_ in one-step MACE always forming Ag dendrites as a byproduct. The activation energy of Si etching for HF/AgO and HF/Ag_2_O are lower smaller than HF/AgNO_3_, being 0.20 eV in HF/AgO etchant [106], and 0.15 eV in HF/Ag_2_O etchant [107]. 

The same MACE techniques used for Si NWs synthesis can also be used for the production of Si microstructures [108], and 3D structures [109].

Si microstructures are easily obtained in a sequence of steps that include selective MACE Si NWs in pre-patterned regions by masked photolithography, which are then sacrificed in an over-etching process leaving the microstructures standing. Si microstructures first synthesis step consists of the patterning realization on Si wafer by photolithography, deposition of noble metal catalyst for MACE in HF/oxidants solution to produce Si nanowires only in the patterned areas, and then etching away the Si nanowires in a KOH-based solution [110]. To allow the MACE processing, noble metal (mainly Ag, Au, and Pt) can be deposited onto the Si surface by different methods which include thermal evaporation [111,112], sputtering [99], electron beam evaporation [79], electroless deposition [80] and spin coating [69]. The whole process can be carried out at room temperature without complex equipment or special facilities, and it can be applicable on a wafer-scale.

## 4. Metal Film or Two Step MACE

In the literature it is possible to find both the name of *metal film* and *thin film* approaches for several types of two step MACE routes. In this paragraph will be presented all the methods reporting the use of metal film in the literature. 

Metal film can be deposited on Si wafer and used as a metal catalyst in a MACE approach. Several works report the use of metal film to offer several advantages in terms of geometry control, diameter, spacing, and density [79,99,112,113,114] compared to MACE approaches that involve layers of nanoparticles similarly to single step MACE (e.g., the silver salts). The metal geometry determines the formation of silicon nanostructures and self-assembly methods can also be used for masking. Most of the approaches take advantage of metal films integration with some masking methods to strictly determine the geometry of the metal array (negative mask of the silicon nanowire array). Indeed, to surpass the lack of control in the Si NWs array geometry, the MACE can be coupled with self-assembly or a lithography process by using ordered metal film as catalysts. This method can be used to realize several silicon nano- and microstructures with a very high aspect ratio, surpassing the limit of 50:1 typical of DRIE. Different groups [64,102] report the use of MACE by high control patterned metal films (usually obtained through EBL) for nano and microfabrication of silicon. This approach can be used to fabricate Si gratings [64], honeycomb array [115] and other microstructures [116,117] or vertical aligned [70,102,113,118], tilted [98,119,120,121], zigzag [122,123,124], or tapered Si nanowires [125].

A typical approach is the one of Miao et al. [115] used to realize a silicon honeycomb array. This array was obtained by depositing the metal in a previously patterned resist, in this case, exposed and developed by UV lithography. After the lift-off, the metal results structured as the negative of the resist mask. After a typical MACE process at room temperature in a HF/H_2_O_2_ bath (H_2_O_2_ 30 wt.%, HF 40wt.%), they obtained a honeycomb array of 50 µm width and 280 µm deep [115].

Another possible approach reported by Li et al. [64] consists of patterning the metal (Ti-Au) through an RIE process. Initially, a 3 nm of Ti as an adhesion layer and 20 nm of Au is deposited by EBE in a Si substrate. The sample is then spun with PMMA and patterned through EBL. The pattern realized through EBL corresponds to the final structure. In fact, the exposed metal is then etched by RIE and then the PMMA is removed. Finally, the sample is immersed in a H_2_O_2_:HF solution with the hydrogen peroxide used as an oxidation agent and a high aspect ratio is obtained with Si structures characterized by a length of 250 nm and a height of 21 µm. 

**Figure 7:**
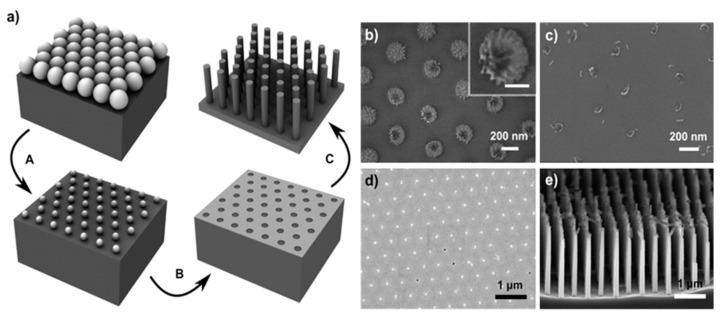
(**a**) Scheme of the Si NWs fabrication by nanosphere lithography coupled with MACE. In particular: (A) nanosphere reduction, (B) lift-off process, and (C) metal-assisted chemical etching. SEM images of the main issues and challenging steps of the process: (**b**) roughened nanosphere due to the RIE etching, in the inset a single roughened nanosphere is shown with a scale bar of 100 nm; (**c**) nanosphere shape ruined after excessive RIE, (**d**) unsuccessfully lift-off of an Au layer, (**e**) tapered and porous Si NWs by MACE [126]. This figure [126] is reproduced with permission, Copyright 2013, John Wiley and Sons.

Yeom et al. report the use of nanosphere lithography for the fabrication of Si NWs through MACE as visible in Figure 7 [126]. In particular, in Figure 7a the schematic of the nanosphere lithography for the realization of the Ti/Au metal film used as a catalyst is shown. After the reduction of the nanosphere dimension by RIE a Ti adhesion layer of a few nm was deposited and then followed by 10–25 nm of Au deposition. In Figure 7b–d, the main possible issues related to the nanosphere etching by RIE are reported. Figure 7b shows that a continuous RIE reduction can increase the roughness of the nanospheres or it can ruin the starting spherical shape (Figure 7c). Another issue is the formation of a continuous metal film (Figure 7d) that completely covers the nanosphere, making the lift-off process very difficult. Finally, in Figure 7e the authors show that as a function of the etching solution main parameters is possible to obtain tapered and porous Si NWs. 

Several types of masking procedures are being used during these years besides the elicited EBL. Wendisch et al. report on the use of colloidal lithography followed by a plasma treatment to reduce the nanosphere size [127]. Kim et al. [113] demonstrate the use of an anodic aluminium oxide mask also used in several other works [128,129]. Other types of diffused masking procedures are polystyrene spheres [112], laser interference lithography [130], superionic solid state stamping [119], and block copolymer lithography [79,131], and even Focused-Ion Beam (FIB) [104].

Despite masked approaches permits to achieve a high control on the Si structure geometry, quantum confined Si NWs remain very complicated to be obtained due to the required resolution of few nanometers. Different authors [12,102] demonstrate the application of MACE without masking approaches. To surpass the common diameter limitations, the group of Irrera engineered a metal film approach by using few nanometers of Au or Ag discontinuous layers. By the percolative self-assembly of the gold obtained at the right deposition condition, this method permits to obtain Si NWs with an average diameter of few nanometers and with a very high density. In this case, no masking procedures are applied and the NWs geometry is determined by the negative development of the percolative gold geometry. 

**Figure 8:**
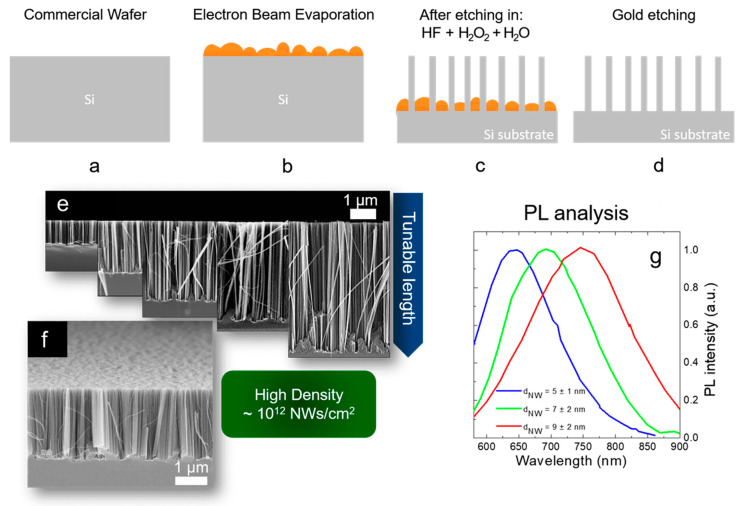
Scheme of Si NW synthesis by thin film MACE: (**a**) native oxide etching, (**b**) thin metal film deposition by EBE, (**c**) metal-assisted chemical etching, (**d**) gold etching. All the processes are performed at room temperature. (**e**) Cross-section SEM images showing the possibility to tune the NW lengths from hundreds of nanometers to several micrometers. (**f**) Tilted Cross-section SEM showing the high NW density of about 10^12^ NWs/cm^2^. Raman analysis of the first order stokes silicon peak. The average Si NW diameter is obtained for each different metal by fitting the Raman peak with the Campbell-Fauchet model [134]. (**g**) Normalized PL spectra of the different NW samples.

As shown in Figure 8a–d, a discontinuous thin metal film is deposited by EBE on an oxide-free Si substrate (Figure 8a,b). The sample is then immersed into a watery solution of H_2_O_2_:HF (around 0.44 M:5 M) with the metal that drives the Si oxidation and so the etching by the HF (Figure 8c). Silicon nanowires are realized on the uncovered region, and the gold is finally removed by a gold etchant solution (Figure 8d). All the processes are performed at room temperature. The presence of the gold is not attested inside the Si NWs by using Energy Dispersive X-ray analysis and Rutherford Backscattering Spectrometry [132,133]. 

As shown in Figure 8e, by changing the etching time the Si NW length varies from a few hundreds of nanometers to several micrometers. Therefore, as visible in Figure 8f the density of the Si NWs is huge (about 10^12^ NWs/cm^2^) and this is a crucial point for all the applications. NW walls are smooth and with high crystalline quality, as previously observed by TEM [132]. The average diameter of these synthesized Si NWs is determined by the thickness and the type of metal used as a catalyst. In fact, the thin metal layer is discontinuous and nanometric areas of uncovered silicon are present. The average dimension of these areas is determined by the material wettability and thickness. By using 10 nm of Ag, 2 nm of Au, and 3 nm of Au an average uncovered Si diameter of 12 nm, 9 nm, and 7 nm, was respectively demonstrated. These data were obtained from the statistical analysis performed on the SEM characterizations of the different films. The dimension of the diameters of these uncovered Si holes is strictly related to the final Si NW average diameters.

In particular, for the different metal thin films, the Si NW average diameters were obtained through Raman analyses. Indeed, the Raman signals of the Si are asymmetrical due to the quantum confinement effect, and fitting them with the Campbell-Fauchet method [134] is possible to obtain the average NW diameter for each used metal. Average diameters of 10 nm, 7 nm, and 5 nm were obtained by using 10 nm of Ag (red line), 2 nm of Au (blue line), and 3 nm of Au (green line), respectively. These diameters were further confirmed by an accurate statistical TEM analysis. These NW diameters are enough to present quantum confinement effect. The emission of all the samples is reported in Figure 8g, demonstrating their quantum confinement nature. In fact, the PL is redshifted by increasing the average diameter of the Si NWs, in perfect agreement with quantum confinement theory [72,135]. 

A possible drawback for this MACE approach is the lack of order. However, the desired geometry that can be obtained by a masking procedure. However, this thin metal film approach is cost-effective to obtain vertically aligned and room temperature luminescent Si NWs.

Different metals were employed in these years as a catalyst during a MACE approach such as Ag [113,132,136,137], Au [64,72,102,115], Pt [104], Ni [103], Cu [123], W [102]. The most used one remains the gold due to the best quality of fabricated Si NWs, for the stability in the etching solution compared to the silver, and for the fast and high tunable etching rate compared to other metals. Ti and other material such as AZO were also used as an adhesion layer for thick Au films, permitting to improve the film stability [126,127,138]. Kim et al. report the application of an Au/Ag bilayer mesh to avoid the issue on the Ag stability and anodic dissolution during the etching [113]. Wendisch et al. used an AZO reporting an improvement in the homogeneity of the etching and the possibility to vary the etching rate by changing its thickness [127]. The same group and also Yeom et al. [126] report gold delamination for high H_2_O_2_ concentrations (e.g., for 10:1:10 HF/H_2_O_2_/H2O) and for in general high etch rates (>400–600 nm/m) and small thin films (<20 nm) [127]. The dependence on the etching rate is confirmed by other works [64] and it is reasonable to assume that metal instability may be also affected by the wafer pre-treatment and deposition type. The etching rate is the key parameter, and a too fast process can be unstable and not affordable in terms of reproducibility. Indeed, in our experiment using few nanometers of Au we never observed gold delamination or large area defects in the Si NW array by an etching rate of 460 nm/min and a solution of HF (5 M) and H_2_O_2_ (0.44 M). The role of a high concentration of H_2_O_2_ is clear because it can increase the hole injection and reaction at the metal silicon interface, thus increasing the bubble formation during the etching possibly affecting the metal stability. These effects and issues regard the use of a connected Au film.

The etching rate is influenced by the gold coverage and so by the NWs diameter. Indeed, the amount of silicon to be etched depends on the covered region of the gold template, on the pitches (average gold region between Si NWs), and on the diameter of Si NWs [127].

NW average diameter is determined by the metal film and usually ranges around 50 nm [102,113] but as seen in Figure 7 with the possibility to reach few nanometers and quantum confinement suitable dimensions [12]. 

The reported preferential etching direction depends on several factors as wafer crystalline orientation, used etchant/oxidant solution, and its molar ratio, temperature, and metal film type and thickness [102,120,122,127,139]. The wafer crystalline orientation has been found as the main parameter that determines the etching orientation with a preferential etching along the <100> direction [90,95,102,140,141]. However, the concentration and ratio between the etchant and oxidant (usually HF/H_2_O_2_) can lead to other orientation etching. This can be understood considering that for a starting crystalline orientation, the etching orientation is driven by the reaction kinetic. Starting from Si (100) it was demonstrated that <100> remain the etching direction at a low etching rate [113]. On the contrary, increasing the etching rate the same authors observed an etching in the <110> direction. The etching rate is characterized by the interplay of holes injection into silicon that is proportional to the metal electronegativity and to the oxidant H_2_O_2_ concentration and by the HF etching of silicon associated with the cleavage of Si back bonds [113]. Indeed, Si atom density exposed to the interface depends on the considered crystal plane [142]. At standard operation conditions, the hole injection is the limiting process of MACE. Increasing the concentration of injected holes above a certain threshold will cause the remotion of Si atoms where there is a higher concentration of Si back bonds causing in certain cases a change in the etching orientation. However, this condition requires a high enough HF concentration and the oxidant concentration as a limiting condition. Otherwise, with a Si (100) wafer, a low concentration of HF slowly etch the silicon in the <100> direction and the excess of holes diffuses causing new pore nucleation sites in presence of defect or doping atoms [102,113]. The temperature plays a role in the activation of non-<100> direction etching. Indeed, considering the same etching solution this can lead to an <100> etching at room temperature and <110> etching at higher temperatures (>50 °C for H_2_O_2_ 1 M and HF/H_2_O_2_ molar ratio of about 23) [113]. Temperature control is also used to low the etching rate and so the possible lateral etching with some works that report on cold MACE synthesis performed near 0 °C [64], while other works used higher temperature above 55 °C to form zigzag Si NWs [122]. 

As elicited, the type of metal mesh plays a role in the etching direction. Several experiments were performed in the same etching condition by using isolated metal particles instead of connected metal films. For metal film the etching is preferentially vertical to the wafer surface due to the difficulty of horizontal movement of the film. On the contrary, metal particles or isolated islands have more degrees of freedom. Indeed, MACE has been used to realize 3D etching taking advantage of the unconstrained metal particles movement during etching at the right conditions [143,144].

Lateral etching or in general a more isotropic etching is found to happen when a large number of holes are injected at the metal-silicon interface [115,145]. The diffusion of these holes can oxidize the sidewall, and lead to a lateral etching and porous nanostructures. The porosity of Si NWs depends on the doping level of Si wafer and on the solution concentration, especially of H_2_O_2_ or other oxidant agents [102]. Heavily doped Si wafers produce more easily porous Si NWs because the doping atoms may act as nucleation centers (e.g., metal atoms in solution), catalyzing the lateral etching and the pore formations [146,147,148]. In highly doped substrates, a competition between vertical etching and lateral etching (and so porous formation) occurs [73,149]. If the vertical etching is fast enough it is possible to obtain crystalline Si NWs at the right bath condition without the formation of porous structure [102,132]. As for the crystalline orientation, there is a strong dependence on the ratio between the H_2_O_2_ (and so hole injection) and HF (and so Si atom remotion) [102].

Concerning the standard HF/H_2_O_2_ Mace bath Kim et al. reported that ethanol can be used to lower the etching rate influencing the etching direction and the final morphology [150]. This can also lead to tapered Si NWs as demonstrate by Azeredo et al. by controlling the ethanol concentration [151]. The addition of other oxidant co-solvent as methanol, isopropanol, acetonitrile, and so on has also been found as a method to change the etching orientation [150]. 

A final consideration is the possible agglomeration of the NWs tips as a result of a very high aspect ratio, and as a function of the drying protocol. Several works reported on the NW tips bunching for very long Si NWs [64,92]. A possible solution proposed in these years is a drying protocol that involves a final bath in isopropanol (lower capillary forces than water) followed by natural evaporation of the alcohol [64,152]. Bunching of micro and nanostructures are typical issues of MEMS. Hence another viable industrial solution for Si NWs or other microstructures is to use a critical point dryer [79,153]. 

Metal film MACE permits a high flexibility of use for Si NW fabrication with several routes that can be followed to change diameter, orientation, density, porous formation, and so on. As demonstrated by the current trend of publications, the MACE method is becoming an outstanding tool for Si NWs synthesis and, for certain applications able to challenge RIE approaches for Si microstructure fabrication. In the next future, this relative novel approach may be really adopted for large-scale production of Si NWs thanks to the advantage of being compatible with the current industrial standard equipment. Indeed, the low-cost of this approach coupled with the very high aspect ratio makes it really appealing for microfabrication, challenging the more diffuse and consolidated approaches as DRIE. 

## 5. Conclusions

In this paper, we presented the strategic role of Si NWs and a focused overview on the Metal-Assisted Chemical Etching (MACE) for the synthesis of Si NWs in comparison to other approaches. Due to the leading position of silicon, the realization of Si nanowires is extremely interesting to face the challenges of the current technology for different applications. In this scenario, the MACE represents a very promising fabrication method for the cost-effective, high-density and large-scale realization of Si NW arrays, obtained in most cases with the current standard microelectronics equipment and with large-scale production. In particular, all the most important MACE routes were investigated critically discussing the advantages and drawbacks starting from the standard silver salt to the ordered and fractal metal film approaches for all of them. Compared to other techniques, MACE offers the perspective of an easy integration with microelectronics technologies at a lower cost with larger processable areas, both disordered and ordered arrays of Si NWs can be obtained tuning different parameters according to the desired applications, enabling the route for the realization of commercial devices based on Si NWs as strategic building blocks.

## References

[1] Waldrop M.M. (2016). More Than Moore. Nature.

[2] Radamson H.H., Zhu H., Wu Z., He X., Lin H., Liu J., Xiang J., Kong Z., Xiong W., Li J. (2020). State of the Art and Future Perspectives in Advanced CMOS Technology. Nanomaterials.

[3] Radamson H.H., He X., Zhang Q., Liu J., Cui H., Xiang J., Kong Z., Xiong W., Li J., Gao J. (2019). Miniaturization of CMOS. Micromachines.

[4] Cobalt Could Untangle Chips’ Wiring Problems—IEEE Spectrum. https://spectrum.ieee.org/semiconductors/materials/cobalt-could-untangle-chips-wiring-problems.

[5] Cui Y., Zhong Z., Wang D., Wang W.U., Lieber C.M. (2003). High performance silicon nanowire field effect transistors. Nano Lett..

[6] Feng W., Hettiarachchi R., Sato S., Kakushima K., Niwa M., Iwai H., Yamada K., Ohmori K. (2012). Advantages of silicon nanowire metal-oxide-semiconductor field-effect transistors over planar ones in noise properties. Jpn. J. Appl. Phys..

[7] Koo S.M., Edelstein M.D., Li Q., Richter C.A., Vogel E.M. (2005). Silicon nanowires as enhancement-mode Schottky barrier field-effect transistors. Nanotechnology.

[8] Garnett E., Yang P. (2010). Light Trapping in Silicon Nanowire Solar Cells. Nano Lett..

[9] Kelzenberg M.D., Boettcher S.W., Petykiewicz J.A., Turner-Evans D.B., Putnam M.C., Warren E.L., Spurgeon J.M., Briggs R.M., Lewis N.S., Atwater H.A. (2010). Enhanced absorption and carrier collection in Si wire arrays for photovoltaic applications. Nat. Mater..

[10] Cao L., Fan P., Vasudev A.P., White J.S., Yu Z., Cai W., Schuller J.A., Fan S., Brongersma M.L. (2010). Semiconductor Nanowire Optical Antenna Solar Absorbers. Nano Lett..

[11] Walavalkar S.S., Hofmann C.E., Homyk A.P., Henry M.D., Atwater H.A., Scherer A. (2010). Tunable visible and near-IR emission from sub-10 nm etched single-crystal Si nanopillars. Nano Lett..

[12] Leonardi A.A., Nastasi F., Morganti D., Lo Faro M.J., Picca R.A., Cioffi N., Franzò G., Serroni S., Priolo F., Puntoriero F. (2020). New Hybrid Light Harvesting Antenna Based on Silicon Nanowires and Metal Dendrimers. Adv. Opt. Mater..

[13] Kalem S., Werner P., Talalaev V. (2013). Near-IR photoluminescence from Si/Ge nanowire-grown silicon wafers: Effect of HF treatment. Appl. Phys. A Mater. Sci. Process..

[14] Leonardi A.A.A.A., Lo Faro M.J.M.J., Di Franco C., Palazzo G., D’Andrea C., Morganti D., Manoli K., Musumeci P., Fazio B., Lanza M. (2020). Silicon nanowire luminescent sensor for cardiovascular risk in saliva. J. Mater. Sci. Mater. Electron..

[15] Patolsky F., Zheng G., Lieber C.M. (2006). Nanowire sensors for medicine and the life sciences. Nanomedicine.

[16] In H.J., Field C.R., Pehrsson P.E. (2011). Periodically porous top electrodes on vertical nanowire arrays for highly sensitive gas detection. Nanotechnology.

[17] Nah J., Liu E.S., Shahrjerdi D., Varahramyan K.M., Banerjee S.K., Tutuc E. (2009). Realization of dual-gated Ge- SixGe1-x core-shell nanowire field effect transistors with highly doped source and drain. Appl. Phys. Lett..

[18] Javey A., Nam S., Friedman R.S., Yan H., Lieber C.M. (2007). Layer-by-layer assembly of nanowires for three-dimensional, multifunctional electronics. Nano Lett..

[19] Goldberger J., Hochbaum A.I., Fan R., Yang P. (2006). Silicon vertically integrated nanowire field effect transistors. Nano Lett..

[20] Lo Faro M.J., Leonardi A.A., Morganti D., Fazio B., Vasi C., Musumeci P., Priolo F., Irrera A. (2018). Low Cost Fabrication of Si NWs/CuI Heterostructures. Nanomaterials.

[21] Liu K., Zhu Z.H., Li X.J., Zhang J.F., Yuan X.D., Guo C.C., Xu W., Qin S.Q. (2015). Bright Multicolored Photoluminescence of Hybrid Graphene/Silicon Optoelectronics. ACS Photonics.

[22] Thomas C.D., Cameron A., Green R.E., Bakkenes M., Beaumont L.J., Collingham Y.C., Erasmus B.F.N., Ferreira De Siqueira M., Grainger A., Hannah L. (2004). Extinction risk from climate change. Nature.

[23] Philipps S., Fraunhofer I.S.E., Warmuth W. (2020). Photovoltaics Report.

[24] Marigo N. (2007). The Chinese silicon photovoltaic industry and market: A critical review of trends and outlook. Prog. Photovolt. Res. Appl..

[25] Neophytou N., Paul A., Klimeck G. (2008). Bandstructure effects in silicon nanowire hole transport. IEEE Trans. Nanotechnol..

[26] Bruno M., Palummo M., Marini A., Del Sole R., Ossicini S. (2007). From Si nanowires to porous silicon: The role of excitonic effects. Phys. Rev. Lett..

[27] Ma D.D.D., Lee C.S., Au F.C.K., Tong S.Y., Lee S.T. (2003). Small-diameter silicon nanowire surfaces. Science.

[28] Guichard A.R., Barsic D.N., Sharma S., Kamins T.I., Brongersma M.L. (2006). Tunable light emission from quantum-confined excitons in TiSi 2-catalyzed silicon nanowires. Nano Lett..

[29] Leonardi A.A., Lo Faro M.J., Petralia S., Fazio B., Musumeci P., Conoci S., Irrera A., Priolo F. (2018). Ultrasensitive Label- and PCR-Free Genome Detection Based on Cooperative Hybridization of Silicon Nanowires Optical Biosensors. ACS Sens..

[30] Giurlani W., Dell’Aquila V., Vizza M., Calisi N., Lavacchi A., Irrera A., Lo Faro M.J., Leonardi A.A., Morganti D., Innocenti M. (2019). Electrodeposition of Nanoparticles and Continuous Film of CdSe on n-Si (100). Nanomaterials.

[31] Chen K.I., Li B.R., Chen Y.T. (2011). Silicon nanowire field-effect transistor-based biosensors for biomedical diagnosis and cellular recording investigation. Nano Today.

[32] Wagner R.S., Ellis W.C. (1964). Vapor-liquid-solid mechanism of single crystal growth. Appl. Phys. Lett..

[33] Wang B., Stelzner T., Dirawi R., Assad O., Shehada N., Christiansen S., Haick H. (2012). Field-effect transistors based on silicon nanowire arrays: Effect of the good and the bad silicon nanowires. ACS Appl. Mater. Interfaces.

[34] Gunawan O., Guha S. (2009). Characteristics of vapor-liquid-solid grown silicon nanowire solar cells. Sol. Energy Mater. Sol. Cells.

[35] Schmidt V., Wittemann J.V., Senz S., Gósele U. (2009). Silicon nanowires: A review on aspects of their growth and their electrical properties. Adv. Mater..

[36] Dubrovskii V.G., Sibirev N.V., Harmand J.C., Glas F. (2008). Growth kinetics and crystal structure of semiconductor nanowires. Phys. Rev. B Condens. Matter Mater. Phys..

[37] Artoni P., Pecora E.F., Irrera A., Priolo F. (2011). Kinetics of si and ge nanowires growth through electron beam evaporation. Nanoscale Res. Lett..

[38] Kodambaka S., Tersoff J., Reuter M.C., Ross F.M. (2006). Diameter-independent kinetics in the vapor-liquid-solid growth of Si nanowires. Phys. Rev. Lett..

[39] Nebol’sin V.A., Shchetinin A.A., Dolgachev A.A., Korneeva V.V. (2005). Effect of the nature of the metal solvent on the vapor-liquid-solid growth rate of silicon whiskers. Inorg. Mater..

[40] Puglisi R.A., Bongiorno C., Caccamo S., Fazio E., Mannino G., Neri F., Scalese S., Spucches D., La Magna A. (2019). Chemical Vapor Deposition Growth of Silicon Nanowires with Diameter Smaller Than 5 nm. ACS Omega.

[41] Cui Y., Lauhon L.J., Gudiksen M.S., Wang J., Lieber C.M. (2001). Diameter-controlled synthesis of single-crystal silicon nanowires. Appl. Phys. Lett..

[42] Hochbaum A.I., Fan R., He R., Yang P. (2005). Controlled growth of Si nanowire arrays for device integration. Nano Lett..

[43] Den Hertog M.I., Rouviere J.L., Dhalluin F., Desré P.J., Gentile P., Ferret P., Oehler F., Baron T. (2008). Control of gold surface diffusion on Si nanowires. Nano Lett..

[44] Kim M.H., Kim I.S., Park Y.H., Park T.E., Shin J.H., Choi H.J. (2010). Platinum assisted vapor-liquid-solid growth of er-si nanowires and their optical properties. Nanoscale Res. Lett..

[45] Koren E., Berkovitch N., Rosenwaks Y. (2010). Measurement of active dopant distribution and diffusion in individual silicon nanowires. Nano Lett..

[46] Gomes D.R., Turkin A.A., Vainchtein D.I., De Hosson J.T.M. (2018). On the mechanism of ion-induced bending of nanostructures. Appl. Surf. Sci..

[47] Puglisi R.A., Garozzo C., Bongiorno C., Di Franco S., Italia M., Mannino G., Scalese S., La Magna A. (2015). Molecular doping applied to Si nanowires array based solar cells. Sol. Energy Mater. Sol. Cells.

[48] Kim K., Park C., Kwon D., Kim D., Meyyappan M., Jeon S., Lee J.S. (2016). Silicon nanowire biosensors for detection of cardiac troponin I (cTnI) with high sensitivity. Biosens. Bioelectron..

[49] Jansen H., Gardeniers H., De Boer M., Elwenspoek M., Fluitman J. (1996). A survey on the reactive ion etching of silicon in microtechnology. J. Micromech. Microeng..

[50] Owen K.J., VanDerElzen B., Peterson R.L., Najafi K. High aspect ratio deep silicon etching. Proceedings of the IEEE International Conference on Micro Electro Mechanical Systems (MEMS).

[51] Yeom J., Wu Y., Selby J.C., Shannon M.A. (2005). Maximum achievable aspect ratio in deep reactive ion etching of silicon due to aspect ratio dependent transport and the microloading effect. J. Vac. Sci. Technol. B Microelectron. Nanom. Struct..

[52] Ghoneim M.T., Hussain M.M. (2017). Highly Manufacturable Deep (Sub-Millimeter) Etching Enabled High Aspect Ratio Complex Geometry Lego-Like Silicon Electronics. Small.

[53] Lasers and Moore’s Law. https://spie.org/news/spie-professional-magazine-archive/2010-october/lasers-and-moores-law?SSO=1.

[54] Ito T., Okazaki S. (2000). Pushing the limits of lithography. Nature.

[55] Harriott L.R. (2001). Limits of lithography. Proc. IEEE.

[56] Solak H.H., Ekinci Y., Käser P., Park S. (2007). Photon-beam lithography reaches 12.5 nm half-pitch resolution. J. Vac. Sci. Technol. B Microelectron. Nanom. Struct..

[57] Totzeck M., Ulrich W., Göhnermeier A., Kaiser W. (2007). Pushing deep ultraviolet lithography to its limits. Nat. Photonics.

[58] Heuberger A., Betz H., Blais P.D. (1983). X-Ray Lithography Using Synchrotron Radiation and Ion-Beam Shadow Printing. Proceedings of the Electron-Beam, X-Ray and Ion-Beam Techniques for Submicron Lithographies II.

[59] Chou S.Y., Krauss P.R., Renstrom P.J. (1996). Imprint lithography with 25-nanometer resolution. Science.

[60] Gates B.D., Xu Q., Stewart M., Ryan D., Willson C.G., Whitesides G.M. (2005). New approaches to nanofabrication: Molding, printing, and other techniques. Chem. Rev..

[61] Vieu C., Carcenac F., Pépin A., Chen Y., Mejias M., Lebib A., Manin-Ferlazzo L., Couraud L., Launois H. (2000). Electron beam lithography: Resolution limits and applications. Appl. Surf. Sci..

[62] Yang J.K.W., Cord B., Duan H., Berggren K.K., Klingfus J., Nam S.-W., Kim K.-B., Rooks M.J. (2009). Understanding of hydrogen silsesquioxane electron resist for sub-5-nm-half-pitch lithography. J. Vac. Sci. Technol. B Microelectron. Nanom. Struct..

[63] Saifullah M.S.M., Ondarçuhu T., Koltsov D.K., Joachim C., Welland M.E. (2002). A reliable scheme for fabricating sub-5 nm co-planar junctions for single-molecule electronics. Nanotechnology.

[64] Li H., Ye T., Shi L., Xie C. (2017). Fabrication of ultra-high aspect ratio (>160:1) silicon nanostructures by using Au metal assisted chemical etching. J. Micromech. Microeng..

[65] Kayes B.M., Filler M.A., Putnam M.C., Kelzenberg M.D., Lewis N.S., Atwater H.A. (2007). Growth of vertically aligned Si wire arrays over large areas (>1 cm2) with Au and Cu catalysts. Appl. Phys. Lett..

[66] Morton K.J., Nieberg G., Bai S., Chou S.Y. (2008). Wafer-scale patterning of sub-40 nm diameter and high aspect ratio (>50:1) silicon pillar arrays by nanoimprint and etching. Nanotechnology.

[67] Nassiopoulou A.G., Gianneta V., Katsogridakis C. (2011). Si nanowires by a single-step metal-assisted chemical etching process on lithographically defined areas: Formation kinetics. Nanoscale Res. Lett..

[68] Li X., Bonn P.W. (2000). Metal-assisted chemical etching in HF/H2O2 produces porous silicon. Appl. Phys. Lett..

[69] Harada Y., Li X., Bohn P.W., Nuzzo R.G. (2001). Catalytic amplification of the soft lithographic patterning of Si. Nonelectrochemical orthogonal fabrication of photoluminescent porous Si pixel arrays. J. Am. Chem. Soc..

[70] Huang Z., Geyer N., Werner P., de Boor J., Gösele U. (2011). Metal-Assisted Chemical Etching of Silicon: A Review. Adv. Mater..

[71] Tsujino K., Matsumura M. (2005). Helical Nanoholes Bored in Silicon by Wet Chemical Etching Using Platinum Nanoparticles as Catalyst. Electrochem. Solid-State Lett..

[72] Leonardi A.A., Lo Faro M.J., Irrera A. (2020). CMOS-Compatible and Low-Cost Thin Film MACE Approach for Light-Emitting Si NWs Fabrication. Nanomaterials.

[73] Chiappini C., Liu X., Fakhoury J.R., Ferrari M. (2010). Biodegradable Porous Silicon Barcode Nanowires with Defined Geometry. Adv. Funct. Mater..

[74] Li S., Ma W., Zhou Y., Chen X., Xiao Y., Ma M., Zhu W., Wei F. (2014). Fabrication of porous silicon nanowires by MACE method in HF/H2O2/AgNO3 system at room temperature. Nanoscale Res. Lett..

[75] Peng K.Q., Yan Y.J., Gao S.P., Zhu J. (2002). Synthesis of large-area silicon nanowire arrays via self-assembling nanoelectrochemistry. Adv. Mater..

[76] Peng K., Fang H., Hu J., Wu Y., Zhu J., Yan Y., Lee S. (2006). Metal-Particle-Induced, Highly Localized Site-Specific Etching of Si and Formation of Single-Crystalline Si Nanowires in Aqueous Fluoride Solution. Chem. A Eur. J..

[77] Donato M.G.M.G., Brzobohatý O., Simpson S.H.S.H., Irrera A., Leonardi A.A.A.A., Lo Faro M.J.M.J., Svak V., Maragò O.M.O.M., Zemánek P. (2019). Optical Trapping, Optical Binding, and Rotational Dynamics of Silicon Nanowires in Counter-Propagating Beams. Nano Lett..

[78] Venkatesan R., Arivalagan M.K., Venkatachalapathy V., Pearce J.M., Mayandi J. (2018). Effects of silver catalyst concentration in metal assisted chemical etching of silicon. Mater. Lett..

[79] Chang S.W., Chuang V.P., Boles S.T., Ross C.A., Thompson C.V. (2009). Densely packed arrays of ultra-high-as pect-ratio silicon nanowires fabricated using block-copolymer lithography and metal-assisted etching. Adv. Funct. Mater..

[80] Peng K., Hu J., Yan Y., Wu Y., Fang H., Xu Y., Lee S., Zhu J. (2006). Fabrication of single-crystalline silicon nanowires by scratching a silicon surface with catalytic metal particles. Adv. Funct. Mater..

[81] Ono S., Oide A., Asoh H. (2007). Nanopatterning of silicon with use of self-organized porous alumina and colloidal crystals as mask. Electrochim. Acta.

[82] Pal A., Ghosh R., Giri P.K. (2015). Early stages of growth of Si nanowires by metal assisted chemical etching: A scaling study. Appl. Phys. Lett..

[83] Nahidi M., Kolasinski K.W. (2006). Effects of Stain Etchant Composition on the Photoluminescence and Morphology of Porous Silicon. J. Electrochem. Soc..

[84] Nahm K.S., Seo Y.H., Lee H.J. (1997). Formation mechanism of stains during Si etching reaction in HF-oxidizing agent-H_2_O solutions. J. Appl. Phys..

[85] Seo Y.H., Nahm K.S., Lee K.B. (1993). Mechanistic Study of Silicon Etching in HF-KBrO_3_-H_2_O Solution. J. Electrochem. Soc..

[86] Huang J.C., Sen R.K., Yeager E. (1979). Oxygen Reduction on Platinum in 85% Orthophosphoric Acid. J. Electrochem. Soc..

[87] Zeis R., Lei T., Sieradzki K., Snyder J., Erlebacher J. (2008). Catalytic reduction of oxygen and hydrogen peroxide by nanoporous gold. J. Catal..

[88] Flätgen G., Wasle S., Lübke M., Eickes C., Radhakrishnan G., Doblhofer K., Ertl G. (1999). Autocatalytic mechanism of H2O2 reduction on Ag electrodes in acidic electrolyte: Experiments and simulations. Electrochim. Acta.

[89] Tsujino K., Matsumura M. (2005). Boring deep cylindrical nanoholes in silicon using silver nanoparticles as a catalyst. Adv. Mater..

[90] Chen C.Y., Wu C.S., Chou C.J., Yen T.J. (2008). Morphological control of single-crystalline silicon nanowire arrays near room temperature. Adv. Mater..

[91] Naffeti M., Postigo P.A., Chtourou R., Zaïbi M.A. (2020). Elucidating the Effect of Etching Time Key-Parameter toward Optically and Electrically-Active Silicon Nanowires. Nanomaterials.

[92] Lo Faro M.J.M.J., Leonardi A.A.A.A., D’Andrea C., Morganti D., Musumeci P., Vasi C., Priolo F., Fazio B., Irrera A. (2020). Low cost synthesis of silicon nanowires for photonic applications. J. Mater. Sci. Mater. Electron..

[93] Cheng S.L., Chung C.H., Lee H.C. (2008). A Study of the Synthesis, Characterization, and Kinetics of Vertical Silicon Nanowire Arrays on (001)Si Substrates. J. Electrochem. Soc..

[94] Chattopadhyay S., Li X., Bohn P.W. (2002). In-plane control of morphology and tunable photoluminescence in porous silicon produced by metal-assisted electroless chemical etching. J. Appl. Phys..

[95] Peng K., Lu A., Zhang R., Lee S.T. (2008). Motility of metal nanoparticles in silicon and induced anisotropic silicon etching. Adv. Funct. Mater..

[96] Peng K., Yan Y., Gao S., Zhu J. (2003). Dendrite-Assisted Growth of Silicon Nanowires in Electroless Metal Deposition. Adv. Funct. Mater..

[97] Salem A.M.S., Harraz F.A., El-Sheikh S.M., Ismat Shah S. (2020). Novel Si nanostructures via Ag-assisted chemical etching route on single and polycrystalline substrates. Mater. Sci. Eng. B Solid-State Mater. Adv. Technol..

[98] Huang Z., Shimizu T., Senz S., Zhang Z., Geyer N., Gösele U. (2010). Oxidation rate effect on the direction of metal-assisted chemical and electrochemical etching of silicon. J. Phys. Chem. C.

[99] Huang Z., Shimizu T., Senz S., Zhang Z., Zhang X., Lee W., Geyer N., Gösele U. (2009). Ordered arrays of vertically aligned [110] silicon nanowires by suppressing the crystallographically preferred <100> etching directions. Nano Lett..

[100] Smith Z.R., Smith R.L., Collins S.D. (2013). Mechanism of nanowire formation in metal assisted chemical etching. Electrochim. Acta.

[101] Weisse J.M., Kim D.R., Lee C.H., Zheng X. (2011). Vertical transfer of uniform silicon nanowire arrays via crack formation. Nano Lett..

[102] Han H., Huang Z., Lee W. (2014). Metal-assisted chemical etching of silicon and nanotechnology applications. Nano Today.

[103] Yue Z., Shen H., Jiang Y., Wang W., Jin J. (2014). Novel and low reflective silicon surface fabricated by Ni-assisted electroless etching and coated with atomic layer deposited Al2O 3 film. Appl. Phys. A Mater. Sci. Process..

[104] Hildreth O., Rykaczewski K., Wong C.P. (2012). Participation of focused ion beam implanted gallium ions in metal-assisted chemical etching of silicon. J. Vac. Sci. Technol. B Nanotechnol. Microelectron. Mater. Process. Meas. Phenom..

[105] Cui L., Xia W.W., Wang F., Yang L.J., Hu Y.J. (2013). Investigations on the Si/SiO2 interface defects of silicon nanowires. Phys. B Condens. Matter.

[106] Kato Y., Adachi S. (2011). Synthesis of Si Nanowire Arrays in AgO/HF Solution and Their Optical and Wettability Properties. J. Electrochem. Soc..

[107] Kato Y., Adachi S. (2012). Fabrication and optical characterization of Si nanowires formed by catalytic chemical etching in Ag_2_O/HF solution. Appl. Surf. Sci..

[108] Kim S.-M., Khang D.-Y. (2014). Bulk Micromachining of Si by Metal-assisted Chemical Etching. Small.

[109] Hildreth O.J., Lin W., Wong C.P. (2009). Effect of catalyst shape and etchant composition on etching direction in metal-assisted chemical etching of silicon to fabricate 3D nanostructures. ACS Nano.

[110] Pérez-Díaz O., Quiroga-González E., Silva-González N.R. (2019). Silicon microstructures through the production of silicon nanowires by metal-assisted chemical etching, used as sacrificial material. J. Mater. Sci..

[111] Fang H., Wu Y., Zhao J., Zhu J. (2006). Silver catalysis in the fabrication of silicon nanowire arrays. Nanotechnology.

[112] Huang Z., Fang H., Zhu J. (2007). Fabrication of silicon nanowire arrays with controlled diameter, length, and density. Adv. Mater..

[113] Kim J., Han H., Kim Y.H., Choi S.H., Kim J.C., Lee W. (2011). Au/Ag bilayered metal mesh as a Si etching catalyst for controlled fabrication of Si nanowires. ACS Nano.

[114] Wang S., Liu H., Han J. (2019). Comprehensive study of Au nano-mesh as a catalyst in the fabrication of silicon nanowires arrays by metal-assisted chemical etching. Coatings.

[115] Miao B., Zhang J., Ding X., Wu D., Wu Y., Lu W., Li J. (2017). Improved metal assisted chemical etching method for uniform, vertical and deep silicon structure. J. Micromech. Microeng..

[116] Zahedinejad M., Farimani S.D., Khaje M., Mehrara H., Erfanian A., Zeinali F. (2013). Deep and vertical silicon bulk micromachining using metal assisted chemical etching. J. Micromech. Microeng..

[117] Li L., Zhang G., Wong C.P. (2015). Formation of Through Silicon Vias for Silicon Interposer in Wafer Level by Metal-Assisted Chemical Etching. IEEE Trans. Compon. Packag. Manuf. Technol..

[118] Zhang M.L., Peng K.Q., Fan X., Jie J.S., Zhang R.Q., Lee S.T., Wong N.B. (2008). Preparation of large-area uniform silicon nanowires arrays through metal-assisted chemical etching. J. Phys. Chem. C.

[119] Chern W., Hsu K., Chun I.S., Azeredo B.P.D., Ahmed N., Kim K.H., Zuo J.M., Fang N., Ferreira P., Li X. (2010). Nonlithographic patterning and metal-assisted chemical etching for manufacturing of tunable light-emitting silicon nanowire arrays. Nano Lett..

[120] Kim J., Kim Y.H., Choi S.H., Lee W. (2011). Curved silicon nanowires with ribbon-like cross sections by metal-assisted chemical etching. ACS Nano.

[121] Sandu G., Avila Osses J., Luciano M., Caina D., Stopin A., Bonifazi D., Gohy J.F., Silhanek A., Florea I., Bahri M. (2019). Kinked Silicon Nanowires: Superstructures by Metal-Assisted Chemical Etching. Nano Lett..

[122] Chen H., Wang H., Zhang X.H., Lee C.S., Lee S.T. (2010). Wafer-scale synthesis of single-crystal zigzag silicon nanowire arrays with controlled turning angles. Nano Lett..

[123] Huang Z.P., Geyer N., Liu L.F., Li M.Y., Zhong P. (2010). Metal-assisted electrochemical etching of silicon. Nanotechnology.

[124] Chen Y., Li L., Zhang C., Tuan C.C., Chen X., Gao J., Wong C.P. (2017). Controlling Kink Geometry in Nanowires Fabricated by Alternating Metal-Assisted Chemical Etching. Nano Lett..

[125] Lin H., Cheung H.Y., Xiu F., Wang F., Yip S., Han N., Hung T., Zhou J., Ho J.C., Wong C.Y. (2013). Developing controllable anisotropic wet etching to achieve silicon nanorods, nanopencils and nanocones for efficient photon trapping. J. Mater. Chem. A.

[126] Yeom J., Ratchford D., Field C.R., Brintlinger T.H., Pehrsson P.E. (2014). Decoupling Diameter and Pitch in Silicon Nanowire Arrays Made by Metal-Assisted Chemical Etching. Adv. Funct. Mater..

[127] Wendisch F.J., Rey M., Vogel N., Bourret G.R. (2020). Large-Scale Synthesis of Highly Uniform Silicon Nanowire Arrays Using Metal-Assisted Chemical Etching. Chem. Mater..

[128] Huang J., Chiam S.Y., Tan H.H., Wang S., Chim W.K. (2010). Fabrication of silicon nanowires with precise diameter control using metal nanodot arrays as a hard mask blocking material in chemical etching. Chem. Mater..

[129] Huang Z., Zhang X., Reiche M., Ltu L., Lee W., Shimizu T., Senz S., Gösele U. (2008). Extended arrays of vertically aligned Sub-10 nm diameter [100] Si nanowires by metal-assisted chemical etching. Nano Lett..

[130] De Boor J., Geyer N., Wittemann J.V., Gösele U., Schmidt V. (2010). Sub-100 nm silicon nanowires by laser interference lithography and metal-assisted etching. Nanotechnology.

[131] Gowrishankar V., Miller N., McGehee M.D., Misner M.J., Ryu D.Y., Russell T.P., Drockenmuller E., Hawker C.J. (2006). Fabrication of densely packed, well-ordered, high-aspect-ratio silicon nanopillars over large areas using block copolymer lithography. Thin Solid Films.

[132] Irrera A., Lo Faro M.J., D’Andrea C., Leonardi A.A., Artoni P., Fazio B., Anna Picca R., Cioffi N., Trusso S., Franzò G. (2017). Light-emitting silicon nanowires obtained by metal-assisted chemical etching. Semicond. Sci. Technol..

[133] Irrera A., Magazzù A., Artoni P., Simpson S.H., Hanna S., Jones P.H., Priolo F., Gucciardi P.G., Maragò O.M. (2016). Photonic Torque Microscopy of the Nonconservative Force Field for Optically Trapped Silicon Nanowires. Nano Lett..

[134] Campbell I.H., Fauchet P.M. (1986). The effects of microcrystal size and shape on the one phonon Raman spectra of crystalline semiconductors. Solid State Commun..

[135] Lo Faro M.J., Leonardi A.A., Priolo F., Fazio B., Miritello M., Irrera A. (2020). Erbium emission in Er:Y2O3 decorated fractal arrays of silicon nanowires. Sci. Rep..

[136] Geyer N., Fuhrmann B., Huang Z., De Boor J., Leipner H.S., Werner P. (2012). Model for the mass transport during metal-assisted chemical etching with contiguous metal films as catalysts. J. Phys. Chem. C.

[137] Li X., Xiao Y., Bang J.H., Lausch D., Meyer S., Miclea P.-T., Jung J.-Y., Schweizer S.L., Lee J.-H., Wehrspohn R.B. (2013). Upgraded Silicon Nanowires by Metal-Assisted Etching of Metallurgical Silicon: A New Route to Nanostructured Solar-Grade Silicon. Adv. Mater..

[138] Wendisch F.J., Abazari M., Mahdavi H., Rey M., Vogel N., Musso M., Diwald O., Bourret G.R. (2020). Morphology-Graded Silicon Nanowire Arrays via Chemical Etching: Engineering Optical Properties at the Nanoscale and Macroscale. ACS Appl. Mater. Interfaces.

[139] Bodo F., Hartmut S.L., Höche H.-R., Schubert L., Werner P., Gösele U. (2005). Ordered Arrays of Silicon Nanowires Produced by Nanosphere Lithography and Molecular Beam Epitaxy. Nano Lett..

[140] Christophersen M., Carstensen J., Rönnebeck S., Jäger C., Jäger W., Föll H. (2001). Crystal Orientation Dependence and Anisotropic Properties of Macropore Formation of p- and n-Type Silicon. J. Electrochem. Soc..

[141] Lehmann V. (1993). The Physics of Macropore Formation in Low Doped n-Type Silicon. J. Electrochem. Soc..

[142] Sze S.M., Ng K.K. (2006). Physics of Semiconductor Devices.

[143] Hildreth O.J., Fedorov A.G., Wong C.P. (2012). 3D spirals with controlled chirality fabricated using metal-assisted chemical etching of silicon. ACS Nano.

[144] Hildreth O.J., Brown D., Wong C.P. (2011). 3D Out-of-Plane Rotational Etching with Pinned Catalysts in Metal-Assisted Chemical Etching of Silicon. Adv. Funct. Mater..

[145] Li L., Zhao X., Wong C.P. (2014). Deep etching of single- and polycrystalline silicon with high speed, high aspect ratio, high uniformity, and 3D complexity by electric bias-attenuated metal-assisted chemical etching (EMaCE). ACS Appl. Mater. Interfaces.

[146] Qu Y., Liao L., Li Y., Zhang H., Huang Y., Duan X. (2009). Electrically conductive and optically active porous silicon nanowires. Nano Lett..

[147] Zhong X., Qu Y., Lin Y.C., Liao L., Duan X. (2011). Unveiling the formation pathway of single crystalline porous silicon nanowires. ACS Appl. Mater. Interfaces.

[148] Balasundaram K., Sadhu J.S., Shin J.C., Azeredo B., Chanda D., Malik M., Hsu K., Rogers J.A., Ferreira P., Sinha S. (2012). Porosity control in metal-assisted chemical etching of degenerately doped silicon nanowires. Nanotechnology.

[149] To W.-K., Tsang C.-H., Li H.-H., Huang Z. (2011). Fabrication of n-Type Mesoporous Silicon Nanowires by One-Step Etching. Nano Lett..

[150] Kim Y., Tsao A., Lee D.H., Maboudian R. (2013). Solvent-induced formation of unidirectionally curved and tilted Si nanowires during metal-assisted chemical etching. J. Mater. Chem. C.

[151] Azeredo B.P., Sadhu J., Ma J., Jacobs K., Kim J., Lee K., Eraker J.H., Li X., Sinha S., Fang N. (2013). Silicon nanowires with controlled sidewall profile and roughness fabricated by thin-film dewetting and metal-assisted chemical etching. Nanotechnology.

[152] Togonal A.S., He L., Roca I Cabarrocas P. (2014). Rusli Effect of wettability on the agglomeration of silicon nanowire arrays fabricated by metal-assisted chemical etching. Langmuir.

[153] Jafri I.H., Busta H., Walsh S.T., Lawton R.A., Miller W.M., Lin G., Ramesham R. (1999). Critical point drying and cleaning for MEMS technology. Proceedings of the MEMS Reliability for Critical and Space Applications.

